# Relationship of Ki-67 index in biopsies of metastatic breast cancer tissue and circulating tumor cells (CTCs) at the time of biopsy collection

**DOI:** 10.1007/s00404-023-07080-y

**Published:** 2023-07-22

**Authors:** Thomas M. Deutsch, Chiara Fischer, Fabian Riedel, Kathrin Haßdenteufel, Laura L. Michel, Marc Sütterlin, Sabine Riethdorf, Klaus Pantel, Markus Wallwiener, Andreas Schneeweiss, Stefan Stefanovic

**Affiliations:** 1 Department of Obstetrics and Gynecology, University of Heidelberg, Im Neuenheimer Feld 440, 69120 Heidelberg, Germany; 2 National Center for Tumor Diseases, Im Neuenheimer Feld 460, 69120 Heidelberg, Germany; 3 Department of Gynecology and Obstetrics, Mannheim University Hospital, University of Heidelberg, Theodor-Kutzer-Ufer 1-3, 68167 Mannheim, Germany; 4 Institute of Tumor Biology, University Hospital Hamburg-Eppendorf, Martinistrasse 52, 20246 Hamburg, Germany; 5 German Cancer Research Center (DKFZ), Im Neuenheimer Feld 280, 69120 Heidelberg, Germany

**Keywords:** Circulating tumor cells (CTC), Ki-67, Metastatic breast cancer (MBC)

## Abstract

**Background:**

The proliferation marker Ki-67 is a major pathological feature for the description of the state of disease in breast cancer. It helps to define the molecular subtype and to stratify between therapy regimens in early breast cancer and helps to assess the therapy response. Circulating tumor cells (CTCs) are a negative prognostic biomarker for progression free (PFS) and overall survival (OS) in patients with metastatic breast cancer. Therefore, the CTC count is often described as surrogate for the tumor burden. Both, decrease of Ki-67 and CTC count are considered as evidence for therapy response. The presented work analyzed the correlation between the Ki-67 indices of metastatic tissue biopsies and CTC counts in biopsy time-adjacent peripheral blood samples.

**Patients and methods:**

Blood samples from 70 metastatic breast cancer patients were obtained before the start of a new line of systemic therapy. CTCs were enumerated using CellSearch® (Menarini Silicon Biosystems, Bologna, Italy) whereas intact CTCs (iCTCs) and non-intact or apoptotic CTCs (aCTCs) were distinguished using morphologic criteria. The proportion of cells expressing Ki-67 was evaluated using immunohistochemistry on biopsies of metastases obtained concurrently with CTC sampling before the start of a new line of systemic therapy.

**Results:**

65.7% of patients had a Ki-67 index of > 25%. 28.6% of patients had ≥ 5, 47.1% ≥ 1 iCTCs. 37.1% had ≥ 5, 51.4% ≥ 1 aCTCs. No correlation was shown between Ki-67 index and iCTC and aCTC count (r = 0.05 resp. r = 0.05, Spearman’s correlation index). High CTC-counts did not coincide with high Ki-67 index. High Ki-67, ≥ 5 iCTCs and aCTCs are associated with poor progression free (PFS) and overall survival (OS).

**Conclusion:**

CTCs and Ki-67 are independent prognostic markers in metastatic breast cancer. High Ki-67 in metastatic tumor tissue is not correlated to high iCTC or aCTC counts in peripheral blood.

## What does this study add to the clinical work

**Table:** 

This study provides information on the prognostic significance of the proliferation marker Ki-67 in biopsies from metastases in advanced breast cancer. It also presents the association with concurrently sampled circulating tumor cells in peripheral blood.	

## Introduction

Despite meaningful improvements in patient survival as a result of new treatment concepts and systemic therapies, metastatic breast cancer (MBC) remains the second leading cause of death amongst women in developed countries [1–4]. Patient-tailored therapies, as well as close monitoring, are essential to achieving adequate treatment response, avoid ineffective therapies, side effects and further evaluate the benefits of new drugs with the ultimate goal of improving both overall survival and quality of life.

The nuclear antigen Ki-67 is expressed in the G1, S and G2 phase of the cell cycle and is a well-established proliferation marker in breast cancer [5]. Ki-67 is a prognostic factor complementing established clinicopathological variables in making treatment decisions in relation to early breast cancer (EBC) [6–8]. In a meta-analysis Petrelli et al. showed that the threshold of > 25% cell Ki-67 staining displays the strongest prognostic significance for overall survival in EBC [9]. The relative proportion of Ki-67 positive cells decreases early during preoperative chemotherapy as a sign of therapy response. Furthermore, the dynamics of Ki-67 across time may indicate treatment response and post-recurrence survival [10–15]. Similar to EBC, expression of Ki-67 in metastatic lesions could be associated with poor overall outcome and reduced disease-free survival [16]. However little data is available on the predictive value of Ki-67 in the metastatic setting [17, 18]. Despite its clinical value for EBC, wide availability and low cost, routine clinical assessment of Ki-67 remains controversial because of low inter-laboratory reproducibility due to a lack of standardized staining, scoring methods as well as validated cut-offs [17, 19].

It has been extensively reported that circulating tumor cells (CTCs) are a reliable predictor of longer overall survival (OS) and progression-free-survival (PFS) in metastatic breast cancer patients [20–27]. CTCs are enumerated by the FDA-approved CellSearch™ system. They are expected to exceed conventional radiographic imaging in regard to precision and reproducibility when it comes to prognosticating survival [28, 29]. Five or more CTCs per 7,5 ml blood at beginning of systemic therapy and/or at any time during therapy are indicative of disease progression and correlate inversely with OS and PFS [25, 30, 31]. Further, a considerable decrease in CTCs is linked to positive response to treatment.

Thus, CTC detection and enumeration should be considered a reliable and cost-effective monitoring instrument [27, 29, 32, 33]. Depending on their morphology, a distinction can be made between intact CTC (iCTC) and non-intact or apoptotic CTC (aCTC) [34–36]. The origin of aCTC is still a matter of discussion. They might be a product of therapy-induced and/or spontaneous apoptosis [37, 38]. On the other hand, continuous presence of aCTCs during systemic therapy in MBC is associated with poor prognosis [39, 40]. Increased tumor proliferation, mapped by an elevated Ki-67 index, also often results in increased cell death in the form of apoptosis [41]. This in turn could promote the release of aCTC into the bloodstream. Therefore, aCTCs might be as well a marker of tumor cell proliferation and correlate to Ki-67 expression of metastatic cancer tissue. Since conventional methods for assessing treatment response are frequently invasive in nature, subject to delay and may fail to detect changes in tumor burden, other specific and sensitive markers are needed to complement traditional monitoring instruments.

The present analysis attempts to highlight the role of CTCs in pathological processes related to breast cancer by means of associating its presence with Ki-67 tumor proliferation index.

## Patients and methods

### Study design and patients

This study was a retrospective, single-center, non-randomized, partially blinded study. The study was conducted at the National Center for Tumor Diseases (NCT), and the Department of Obstetrics and Gynecology, University of Heidelberg, Germany. All patients provided written informed consent. The Ethical Committee of the Medical Faculty of the University of Heidelberg approved this study, approval No. S-295/2009.

Between March 2010 and April 2018, all patients ≥ 18 years suffering from metastatic breast cancer were assessed for study participation eligibility. Inclusion criteria comprised the initiation of a new line of treatment regardless of interval since the initial diagnosis, diagnosis of metastasis or previous treatment. Further, Ki-67 proliferation index determined 60 days prior to inclusion was a premise for participation, as was the availability of a blood sample for CTC enumeration at the time of enrollment. Patients lost to follow-up were excluded.

Most biopsies and immunohistochemical analyses were performed at Heidelberg University. In cases where the biopsy was carried out elsewhere, the results of immunohistochemical staining and histology were obtained from medical records of the respective institution.

Clinical documentation was collected retrospectively based on pre-existing medical records. CTC analyses were performed at Heidelberg University Hospital and the Institute for Tumor Biology at Hamburg-Eppendorf University Hospital. Treating physicians, investigators and technical staff involved in collecting data were unaware of patients’ CTC status, history as well as treatment, respectively. Hence, in no manner did therapeutic decisions relate to CTC status. Independent reviewers confirmed CTC enumeration and characterization.

### Enumeration of circulating tumor cells (CTCs)

Peripheral blood was collected upon recruitment to determine baseline total CTC counts of intact CTC and apoptotic CTC counts. Enumeration of the CTCs was performed using the CellSearch™ assay (CellSearch™ Epithelial Cell Kit/CellSpotter™ Analyzer, Menarini Silicon Biosystems, Bologna, Italy), which provides high intra- and inter-instrument accordance. Detailed descriptions of the assay are published by Riethdorf et al. 2007 [42]. Samples with ≥ 5 CTC per 7.5 ml of blood were considered CTC positive [24, 43].

### Ki-67 in metastases

Ki-67 data were taken from pathology reports. The reported Ki-67 index was the average reported as the percentage of nuclear staining-positive cells in immunohistochemical (IHC) tissue blocks. The ≥ 25% threshold was chosen as the distinguishing criterion between high and low Ki-67 [9].

### Survival

Metastatic sites were evaluated by standard imaging techniques. Tumor burden was monitored every three months and classified as progressive disease (PD), stable disease (SD), complete remission (CR), or partial response (PR) according to RECIST 1.1 guidelines [44]. Neither laboratory staff nor independent reviewers had access to relevant clinical data. Survival status, including both overall survival and disease-free survival, was recorded until death and/or end of follow-up.

### Statistical analysis

Demographic data were analyzed and presented as frequencies, means, median, confidence intervals and standard deviations. Survival time was assessed in months from time of enrollment until recurrence of disease (progression-free survival, PFS) and/or death by any cause (overall survival, OS). Data was censored at last follow-up (March 2022) in the case that no such event had occurred. The correlations between survival on one hand and CTCs and Ki-67 expression on the other was evaluated by means of Kaplan–Meier as well as logistic regression analysis. Spearman-correlation was utilized in order to analyze the correlations between iCTC and aCTC counts and Ki-67-indices. Said correlation is possibly subject to the following confounding variables: elapsed time between initial biopsy and blood sampling, differences in therapeutic interventions, organs subject to metastasis, hormone receptor as well as HER2 growth hormone receptor status as well as instances of systemic therapy such as endocrine therapy, chemotherapy, new therapeutics and/or HER2 antibodies.

Statistical analysis was performed using R version 3.5.1 [45]. An alpha significance level of 5% was chosen.

## Results

### Patient characteristics

The clinical and histopathological characteristics of the 70 patients included in the study are listed in Table 1. The median interval between metastatic biopsy and CTC enumeration was 33 days with a maximum of 58 days. Ten patients (14.3%) had metastases at initial breast cancer diagnosis. Most common biopsied sites were liver (25/70, 35.7%), skin/soft tissue (18/70, 25.7%), lymph nodes (9/70, 12.9%) and pleura (6/70, 8.6%). Follow-up data were available for a median of 25 months (1–118 months).

**Table 1:** Clinical and histopathological characteristics of patients included depending on Ki-67 index

Variable	Ki-67 index in MBC cells			
	< 25%	≥ 25%	Total	*p*
n (%)	24 (34.3)	46 (65.7)	70	
Age at initial diagnosis, *mean (SD)*	55.8 (13.1)	50.3 (11.0)	52.1 ( 11.9)	**0.007**
Hormone receptor status primary tumor, *n* (%)			65	**0.01**
Positive (ER)	19 (90.5)	26 (59.1)		
HER2-status primary tumor, n (%)			61	1
Positive	3 (16.7)	9 (20.9		
Triple negative primary tumor, n (%)	1 (4.8)	11 (25.0)		0.08
Grading primary tumor, n (%)			58	0.13
G1/G2	12 (66.7)	18 (45.0)		
G3	6 (33.3)	22 (55.0)		
Hormone receptor status metastasis, *n* (%)			67	0.08
Positive (ER)	19 (82.6)	27 (61.4)		
HER2-status metastasis, *n* (%)			66	0.35
Positive	3 (13.0)	11 (25.6)		
Triple negative metastasis, n (%)	1 (4.3)	11 (25.0)		**0.05**
Metastatic sites, *n* (%)			67	
Other visceral organs	0 (0)	3 (6.7)		0.55
Brain	0 (0)	2 (4.4)		1
Soft tissue/skin	2 (9.1)	10 (22.2)		0.31
Bone	1 (4.5)	0 (0)		0.33
Lymph node	1 (4.5)	8 (17.8)		0.25
Liver	11 (50.0)	14 (31.1)		0.18
Lung	2 (9.1)	3 (6.7)		1
Pleura	2 (9.1)	2 (4.4)		0.59
Thoracic wall	3 (13.6)	3 (6.7)		0.39
Therapy lines, *mean (SD)*	1.96 (2.0)	1.66 (0.48)	70	0.56
Therapy regiments before inclusion, *n* (%)				
Chemotherapy	9 (50.0)	23 (53.5)	61	0.8
Endocrine therapy	10 (35.7)	18 (64.3)	70	0.84
Chemo- and endocrine therapy	5 (27.8)	11 (25.6)	61	1
HER2 targeted therapy	3 (12.5)	10 (21.7)	70	0.52
Therapy regiments after inclusion, n (%)				
Chemotherapy	15 (62.5)	26 (56.5)	70	0.63
Endocrine therapy	10 (41.7)	18 (39.1)	70	0.84
HER2 targeted therapy	2 (3.4)	8 (17.4)	70	0.48
Mean PFS	17.6 ± 20.4	6.5 ± 8.7	65	**< 0.001**
Median PFS	9 (2–70)	3 (0–46)		
Mean OS	36.1 ± 30.8	27.6 ± 25.5	70	0.13
Median OS		27.5 (7 – 118)	3 (0 – 46)	

*p *≤ 0.05

### Ki-67 index

Mean Ki-67 index of metastatic lesions was 41.1%. The mean Ki-67 indices of the most frequent biopsied metastatic sites were: 35.6% in liver metastases, 44.4% in skin/soft tissue metastases, 57.2% in lymph node metastases, and 34.2% in pleural metastases. Low Ki-67 index of metastatic tissue was significantly correlated with ER positive primary tumors (p = 0.01).

### CTC counts

33 (47.1%) patients had ≥ 1 iCTCs and 36 (51.4%) patients had ≥ 1 aCTCs. 20 (28.6%) patients were iCTC-positive, i.e., ≥ 5 iCTCs, 26 (37.1%) were aCTC-positive, i.e., ≥ 5 aCTCs. The patients had mean of 14 aCTCs per 7.5 ml of blood ranging from 0 to 1019 iCTCs/7.5 ml and a of 13 aCTCs per 7.5 ml of blood ranging from 0 to 240 aCTCs/7.5 ml.

### Ki-67 index and CTC status

The Ki-67 index in metastatic biopsies correlated (Spearman’s correlation) neither with the number of iCTCs (p = 0.67) nor aCTCs (p = 0.67) found in blood at the time of initiating novel systemic therapy. Logistic regression modeling did not show significant correlations between the Ki-67 index and iCTC or aCTC status (p_iCTC/Ki-67_ = 0.9, p_aCTC/Ki-67_ = 0.29, respectively). The correlation between Ki-67 index and iCTC and aCTC status, respectively, was not significantly affected by any of the following factors as per our logistic regression: interval from metastasis biopsy to CTC enumeration, line of therapy, metastatic sites, hormone receptor and HER2 status of metastasis and therapy regimen (endocrine therapy, chemotherapy, new therapeutics, HER2 antibody therapy) as depicted in Table 2.

**Table 2:** Cox regression analysis for Survival

Variable	N	Univariate OS		Multivariate OS	
		HR (95% CI)	p	HR (95% CI)	p
Time between initial diagnosis and metastasis		1.51 (0.73–3.12)	0.27	1.15 (0.17 – 7.93)	0.89
≤ 60 months	45				
> 60 months	19				
Axillary lymph node status of the primary tumor		0.8 (0.42 – 1.52)	0.5	0.96 (0.24 – 3.77)	0.95
Negative	22				
Positive	44				
Recurrence Free Survival		0.92 (0.09 – 0.55)	0.01	0 (0 – > 1000)	0.96
≤ 24 months	58				
> 24 months	12				
Estrogen receptor status of the primary tumor		1.71 (0.88 – 3.32)	0.12	1.15 (0.39 – 5.06)	0.81
Negative	20				
Positive	45				
HER2 receptor status of the primary tumor		0.83 (0.39 – 1.79)	0.63	0.84 (0.36 – 3.64)	0.83
Negative	49				
Positive	12				
Estrogen receptor status of the metastasis		0.4 (0.05 – 3.20)	0.39		
Negative	46				
Positive	21				
HER2 receptor status of the metastasis		2.02 (0.58 – 6.70)	0.27		
Negative	52				
Positive	14				
Adjuvant/neoadjuvant chemotherapy		0.79 (0.39 – 1.58)	0.5	0.59 (0.16 – 4.39)	0.73
No	18				
Yes	52				
Metastatic site		0.68 (0.3 – 1.53)	0.35	1.15 (0.32 – 21.95)	0.93
Visceral	60				
Non-visceral	10				
iCTC		0.74 (0.23 – 2.41)	0.62		
< 1	37				
≥ 1	33				
aCTC		0.78 (0.25 – 2.38)	0.66		
< 1	34				
≥ 1	36				
iCTC status		1.51 (0.31 – 7.33)	0.61		
< 5	51				
≥ 5	19				
aCTC status		0.81 (0.17 – 3.9)	0.79		
< 5	44				
≥ 5	26				
Ki-67 status of the primary tumor		0.97 (0.40 – 2.30)	0.94	0.37 (0.57 – 2.42)	0.3
< 25%	10				
≥ 25%	24				
Ki-67 status of the metastasis		2.28 (0.62 – 8.41)	0.22		
< 25%	24				
≥ 25%	46				
Ki-67 status of the metastasis + iCTC status		3.01 (0.39 – 23.5)	0.29		
< 25% + < 5	18				
≥ 25% + ≥ 5	13				
Ki-67 status of the metastasis + aCTC status		2.11 (0.28 – 16.15)	0.47		
< 25% + < 5	16				
≥ 25% + ≥ 5	18				

### Survival analysis

**Fig. 1:**
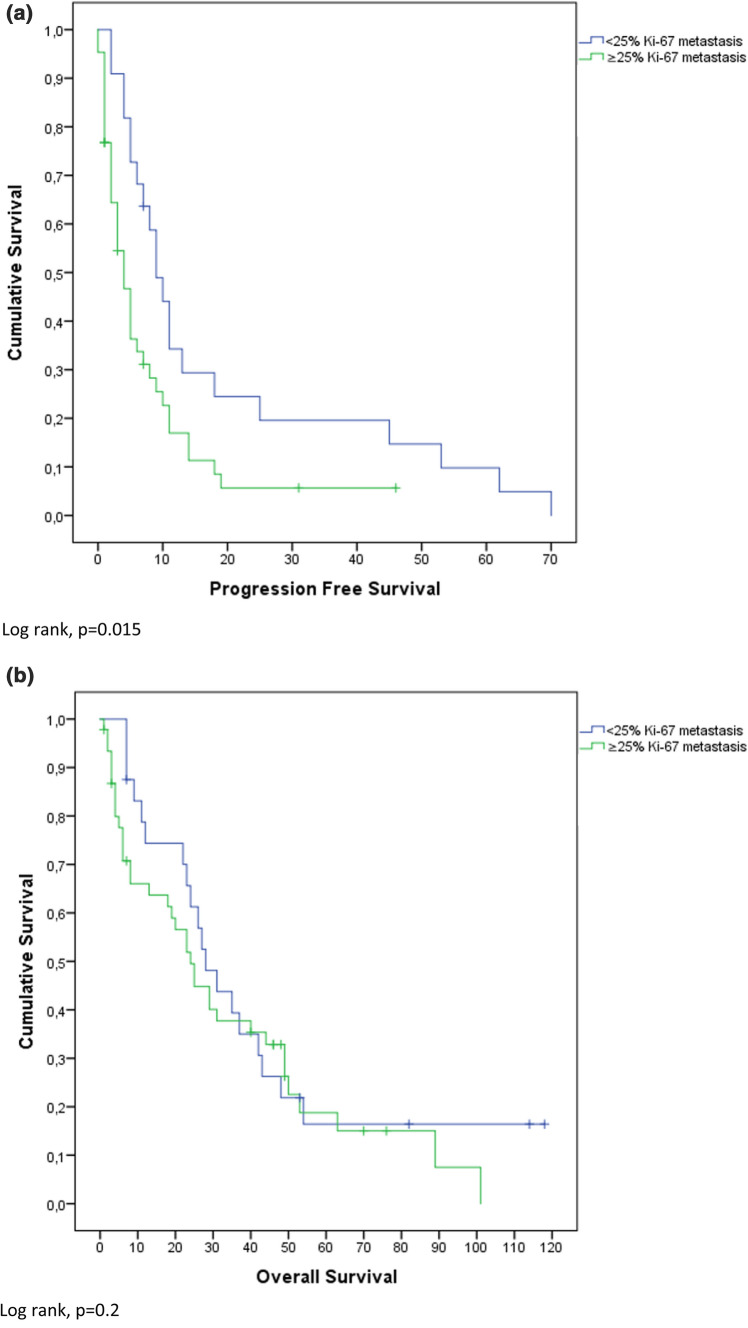
Kaplan–Meier plots representing differences in (**a**) PFS (in months) and (**b**) OS (in months) between groups with a cut-off of < 25% Ki-67 index in metastatic tissue

**Fig. 2:**
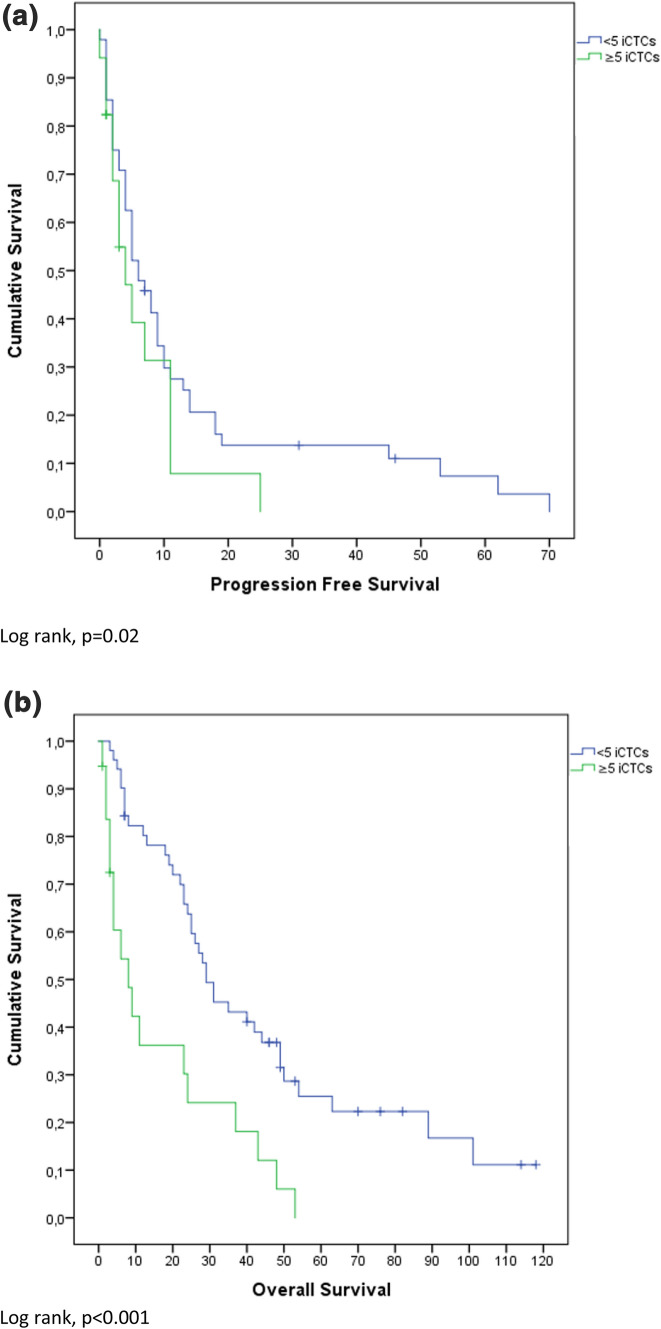
Kaplan–Meier plots representing differences in (**a**) PFS (in months) and (**b**) OS (in months) between groups with a cut-off of ≥ 5 iCTCs

**Fig. 3:**
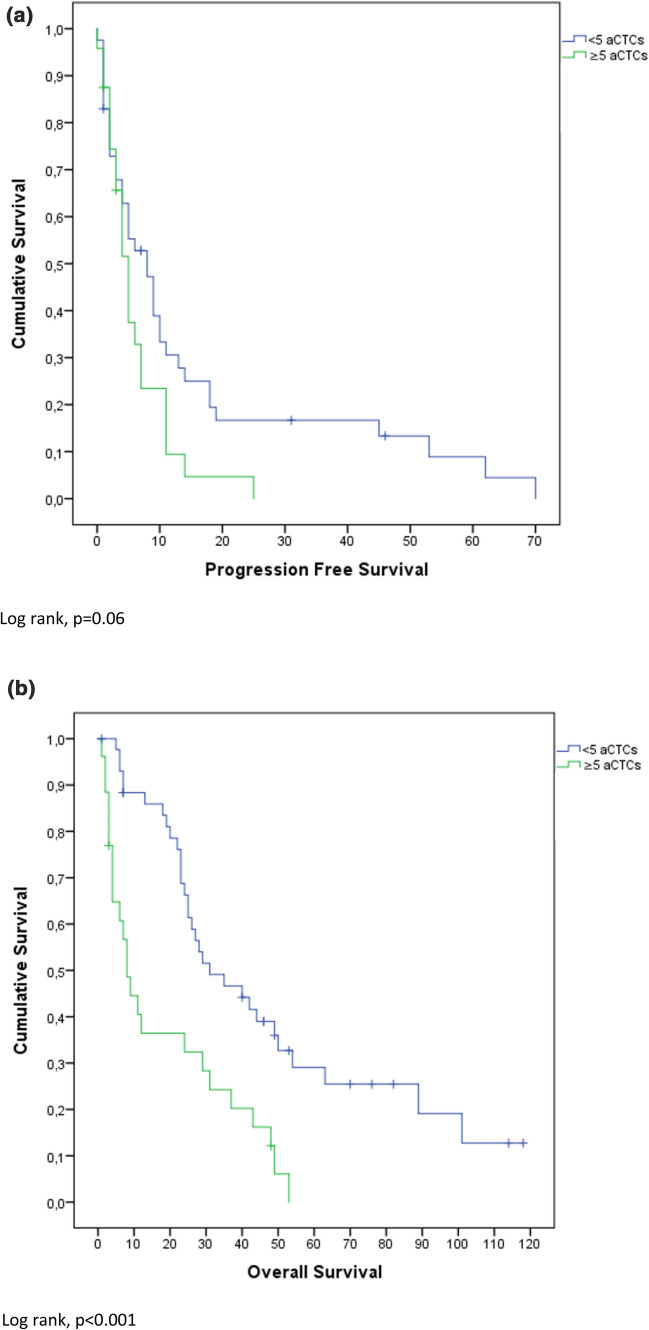
Kaplan–Meier plots representing differences in (**a**) PFS (in months) and (**b**) OS (in months) between groups with a cut-off of ≥ 5 aCTCs

**Fig. 4:**
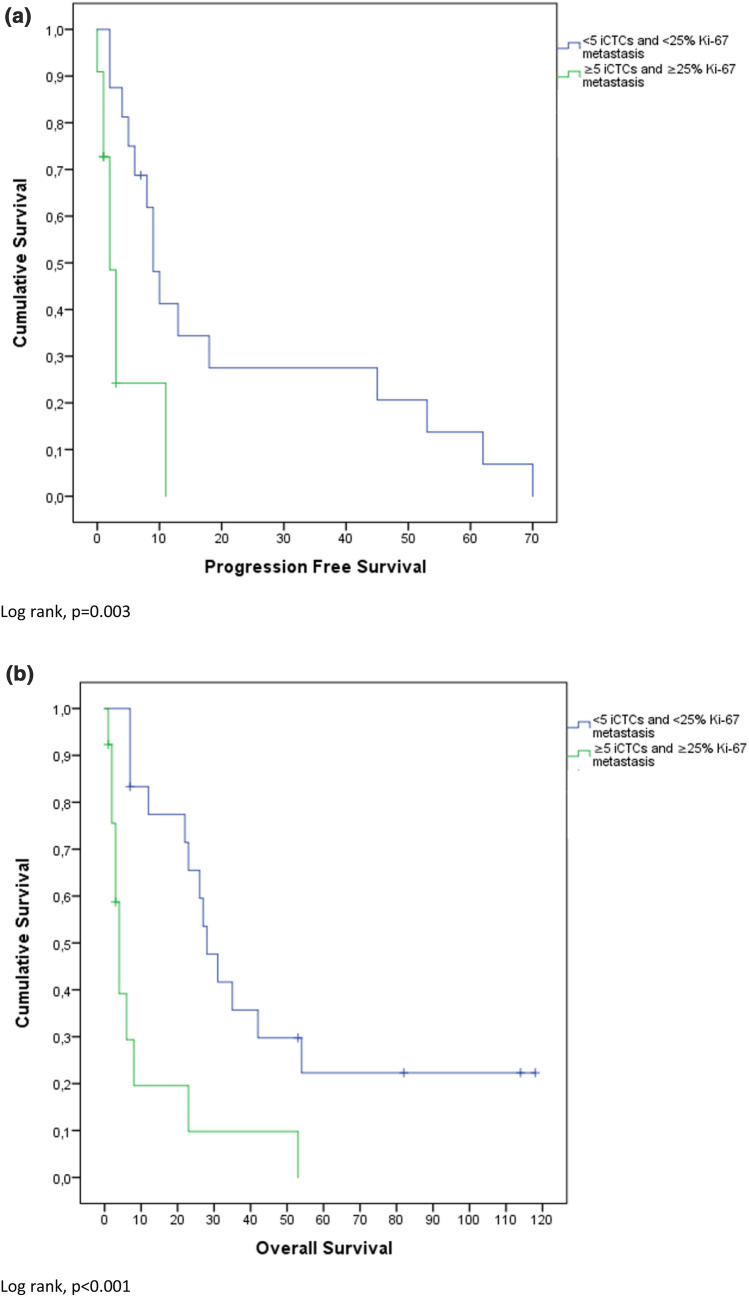
Kaplan–Meier plots representing differences in (**a**) PFS (in months) and (**b**) OS (in months) between groups with < 5 iCTCs and Ki-67 index < 25% vs. ≥ 5 iCTCs and Ki-67 index ≥ 25%

**Fig. 5:**
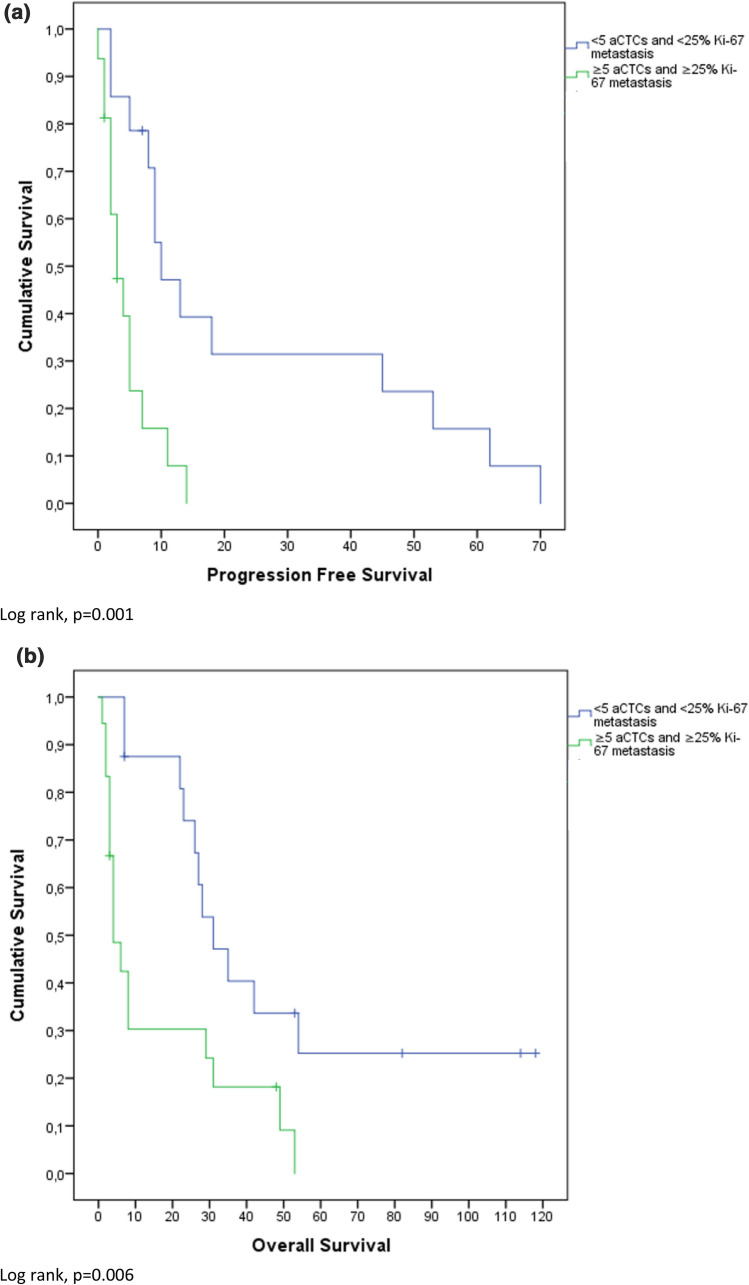
Kaplan–Meier plots representing differences in (**a**) PFS (in months) and (**b**) OS (in months) between groups with < 5 iCTCs and Ki-67 index < 25% vs. ≥ 5 aCTCs and Ki-67 index ≥ 25%

Mean OS was 30.5 months (confidence interval (CI) 3.0–58.0 months) and PFS was 10.2 months (CI 0–24.8 months). High Ki-67 index (≥ 25%) was significantly negatively associated with PFS (p < 0.001). Overall survival (OS) was not significantly different between high and low Ki-67 expressors (p = 0.13). These results are also depicted in Fig. 1. Figure 2 shows that ≥ 5 iCTCs was significantly associated with shorter OS and PFS (p < 0.001; p = 0.02). Also ≥ 5 aCTCs correlated with shorter OS and PFS (p < 0.001; p = 0.06) as depicted in Fig. 3. Figures 4 and 5 reveal the prognostic value of the combination of a negative iCTC or aCTC status (i.e., < 5 CTCs) and a low Ki-67 index (i.e., < 25%). PFS and OS were significantly better for negative iCTC status and low Ki-67 (p = 0.003; p < 0.001) and for negative aCTC status and low Ki-67 (p = 0.001; p = 0.006). Multivariate cox regression analysis (Table 2) revealed no information with further consequences for survival.

## Discussion

Breast cancer is a very heterogeneous and consistently evolving disease, which requires close monitoring of systemic tumor burden and treatment efficacy to guide therapy comprehensively. Sequential biopsies are invasive, not always feasible and represent the progression dynamics in the particular lesion instead of the entirety of the tumor burden. Therefore, focus has increasingly shifted towards liquid biopsies, as minimal-invasive, reliable and cost-effective methods to determinate tumor composition and characteristics, such as, for example, the characterization of CTCs and cell-free DNA (cfDNA) in peripheral blood samples. Circulating tumor cells are a well-known negative prognostic marker in relation to overall and progression-free survival. Further, evidence suggests that the number of CTCs is a good estimate of tumor burden, and real-time monitoring of CTC counts may be used to improve clinical management [28, 32, 33, 37, 46]. Given that CTC count increases with tumor progression, the number of CTCs may correlate with proliferation rates (represented by Ki-67 indices) of metastatic sites. The present study set out to analyze the relationship between the Ki-67 indices of relevant metastatic tissues and CTC counts in biopsy time-adjacent peripheral blood samples.

In line with other studies, the Ki-67 proliferation index was significantly positively correlated with unfavorable outcomes. A high Ki-67 index was associated with poor overall and progression-free survival, which might reflect the aggressiveness of the tumor and therefore could be considered as a factor in the choice of further therapeutic treatment [17]. In addition, the Ki-67 index correlated significantly with the estrogen, progesterone and HER2 growth hormone receptor status which are also prognostic markers for overall survival and play a key role in therapy decisions. However, in contrast to these markers, the Ki-67 index offers the possibility to represent the current local tumor cell proliferation as a sign of response to therapy. This underlines the important clinical role of the Ki-67 index as a prognostic marker and guide for therapeutic decisions in breast cancer patients.

Contrary to our initial hypothesis, we found no significant correlation between the number of intact and/or apoptotic CTCs and Ki-67 proliferation indices in metastatic tissues. Neither lines of therapy, therapeutic regimens, organs affected nor hormone receptor or HER2 growth hormone receptor status influenced said correlation.

The Ki-67 index might only represent the proliferative activity within a single metastatic site and sometimes only within a section of a metastasis. Small (core-needle) biopsies are unlikely to reflect the global proliferative activity of a given tumor, which might cause an under- or overestimation of global tumor proliferation rate. In addition, the most easily accessible progressive metastases are usually biopsied (i.e. liver or skin/soft tissue), and again, this may not represent the entire disease. Aside from the proliferative activity of the tumor itself, there are other factors related to the dissemination of CTCs into the blood stream [47]. For example, epithelial-mesenchymal transition facilitates the spread of CTCs by increasing the expression of genes associated with migration, invasiveness and intravasation of cells into the bloodstream [48]. Furthermore, the microenvironment of the tumor, as represented by chemokines and immune cells, could play an important role in the release of iCTCs.

Overall, patients with positive CTCs status had an inferior outcome in comparison to patients with negative CTCs status. OS and PFS were significantly shorter in patients with ≥ 5 iCTCs (p < 0.001; p = 0.02) and also ≥ 5 aCTCs showed shorter survival for OS and PFS (p < 0.001; p = 0.06). This theory is further supported by the extensive evidence of the prognostic properties of iCTCs published in current literature. The combination of CTC and Ki-67 status as depicted in Figs. 4 and 5 might help to further stratify between stage IV indolent and aggressive cases [22]. In CTC negative cases, Ki-67 index of the metastasis could provide additional prognostic information.

Our study showed that the proliferative activity in metastatic tissues as determined by Ki-67 analysis is not statistically significantly associated with the overall number of CTCs in peripheral blood. Nevertheless, Ki-67 indices and CTCs are prognostic markers in MBC patients. As a combination, they provide valuable prognostically information in the metastatic setting. Future studies are needed to elucidate mechanisms that influence dynamic release of CTCs into the bloodstream in order to advance the understanding of the biology of CTCs and the implications for their clinical application and appreciation.

### Limitations

Limitations of our study include a retrospective design and decentralized determination of Ki-67 indices. Thus, for patients treated at different hospitals, immunohistochemical analysis was performed in non-standardized laboratory settings. Further, Ki-67 indices of relevant biopsies have to be interpreted with caution, because of its limited inter-laboratory reproducibility due to missing standardized staining, scoring methods and consistent cut-offs [17, 19]. Other studies divided Ki-67 index into a high and a low risk group with a 20% cut-off [18]. The percentage composition of the examined metastases in this study does not reflect the most frequent metastasis localities in MBC. In particular, bone metastases are underrepresented. However, the biopsy and histological workup of bone metastases is associated with a significantly greater effort for the patient and the examiner and is therefore less frequently practiced in clinical routine. Also, the sensitivity of the Cell-Search System is limited. CellSearch is an EpCAM based detection method, which might underestimate CTC counts when EpCAM/epithelial-markers on CTCs are downregulated by epithelial-mesenchymal-transition.

## Data Availability

The data that support the findings of this study are not openly available due to reasons of sensitivity and are available from the corresponding author upon reasonable request.
